# Suicidality as a Predictor of Overdose among Patients with Substance Use Disorders

**DOI:** 10.3390/jcm11216400

**Published:** 2022-10-29

**Authors:** Viviana E. Horigian, Renae D. Schmidt, Dikla Shmueli-Blumberg, Kathryn Hefner, Judith Feinberg, Radhika Kondapaka, Daniel J. Feaster, Rui Duan, Sophia Gonzalez, Carly Davis, Rodrigo Marín-Navarrete, Susan Tross

**Affiliations:** 1 Department of Public Health Sciences, University of Miami Miller School of Medicine, 1120 Northwest 14th Street, Miami, FL 33136, USA; 2 The Emmes Company, LLC, 401 N. Washington St., Suite 700, Rockville, MD 20850, USA; 3 Departments of Behavioral Medicine and Psychiatry & Medicine/Infectious Diseases, West Virginia University School of Medicine, 930 Chestnut Ridge Road, Morgantown, WV 26505, USA; 4 Division of Research and Translational Education, Centros de Integración Juvenil A.C., San Jerónimo Avenue 372, Jardines del Pedregal, Mexico City 01900, Mexico; 5 Department of Psychiatry, Columbia University, 1051 Riverside Drive, New York, NY 10032, USA

**Keywords:** substance use disorders, dual disorders, co-occurring disorders, suicidality, overdose

## Abstract

Increasing rates of overdose and overdose deaths are a significant public health problem. Research has examined co-occurring mental health conditions, including suicidality, as a risk factor for intentional and unintentional overdose among individuals with substance use disorder (SUD). However, this research has been limited to single site studies of self-reported outcomes. The current research evaluated suicidality as a predictor of overdose events in 2541 participants who use substances enrolled across eight multi-site clinical trials completed within the National Drug Abuse Treatment Clinical Trials Network between 2012 to 2021. The trials assessed baseline suicidality with the Concise Health Risk Tracking Self-Report (CHRT-SR). Overdose events were determined by reports of adverse events, cause of death, or hospitalization due to substance overdose, and verified through a rigorous adjudication process. Multivariate logistic regression was performed to assess continuous CHRT-SR score as a predictor of overdose, controlling for covariates. CHRT-SR score was associated with overdose events (*p* = 0.03) during the trial; the likelihood of overdose increased as continuous CHRT score increased (OR 1.02). Participants with lifetime heroin use were more likely to overdose (OR 3.08). Response to the marked rise in overdose deaths should integrate suicide risk reduction as part of prevention strategies.

## 1. Introduction

Increasing rates of overdose and overdose deaths are significant public health problems in the US [1]. While opioids have been the leading cause of overdose and overdose deaths, recent evidence suggests increases in overdose deaths due to stimulants [1,2]. Studies also suggest that among individuals who use substances, concurrent use of multiple substances is “…the norm rather than the exception” [3]. Research also indicates that individuals might shift their substance use preferences across their lifespan [4]. It is important to understand and address the social determinants of health and to identify factors and underlying conditions that put individuals at risk for overdose and other adverse outcomes.

The frequent co-occurrence of mental health conditions among individuals with substance use disorder (SUD) is often termed “dual disorders” [5]. Mental health conditions are highly prevalent in individuals seeking treatment for substance use disorders [6]. Major depression (50–60%) [7,8], post-traumatic stress disorder (PTSD) (47%) [9], and anxiety (31.2%) [10] are common among persons with opioid use disorder (OUD), and a majority report suffering from insomnia [11]. Similarly, mental health conditions are highly prevalent in persons with stimulant use disorders, with 35.7 to 41.6% having a lifetime history of major depression [8] and between 23 to 42% with lifetime history of PTSD [12]. Depressive symptoms along with other mental health conditions have been associated with nonfatal overdoses among individuals with SUD, drawing attention to the importance of early identification and treatment for these co-occurring conditions [6,13,14].

Suicide is the 10th leading cause of death in the United States and is a contributor to premature mortality [15]. With the goal of better understanding and preventing opioid overdose and overdose fatalities, recent literature has drawn attention to the distinction between intentional and unintentional overdoses among opioid users [2,16]. Suicidal thoughts might increase the risk of non-fatal overdose and potentially elevate the risk for future intentional overdose or unintentional overdose. Because suicidal ideation and intent may underlie many overdose events [17], studies have shed light on the importance of further characterizing overdose events with the final goal of deploying specific prevention strategies to individuals with suicidal risk and intent [16,18,19]. While these are important contributions that have brought attention to suicidality as an overdose risk factor, these analyses have been limited to single-site or single system studies, have focused on small samples of OUD patients with self-reported intentionality and outcomes, and have been constrained by patient recall.

The co-occurrence of mental health and substance use disorders increases suicidal ideation and behavior [20]. The identification of suicidality is therefore clinically relevant, particularly among persons with dual disorders [21,22,23]. The Concise Health Risk Tracking SR (CHRT-SR) [24] is a self-reported measure that—unlike other clinical assessments for suicide [25,26]—assesses other important associated symptoms related to suicide propensity aside from ideation and intent. These include pessimism, lack of social support, helplessness, and despair. The CHRT-SR has proven to have excellent psychometric properties in patients with major depression [24], bipolar disorders [27,28], and stimulant use disorders [29]. Early identification at treatment entry, whether the individual is driven by suicidal thoughts and intent or by hopelessness and despair, expands the opportunities for intervention and could prevent fatalities.

The objective of this study is to evaluate whether suicidality at treatment entry is a baseline predictor of overdose events in patients with SUD, using data from the National Drug Abuse Treatment Clinical Trials Network (CTN) [30] multi-protocol platform and its associated NIDA Data Share website. We predict that those with higher baseline suicidality assessment scores, indicative of suicidal propensity, ideation, and/or intent, will be more likely to have an overdose event than those with lower scores.

## 2. Methods

The study uses data collected from eight randomized clinical trials that were implemented within the CTN, a network that provides an infrastructure in which the National Institute on Drug Abuse (NIDA), medical and specialty treatment providers, academic centers, researchers, and patients cooperatively develop, validate, refine, and deliver new treatment options for patients with SUD [30]. There are 16 CTN research sites termed ‘nodes’ in the U.S. that conduct clinical trials across diverse settings and populations. For this study, we analyzed data that was approved for public release or that was publicly available on NIDA Data Share website (https://datashare.nida.nih.gov/ (accessed on 15 February 2022)). Data Share is an electronic environment that allows access to data from completed trials to promote new research using secondary analyses [31].

### 2.1. Participants

We analyzed data from eight multicenter CTN trials that included 2543 participants [32,33,34,35,36,37,38,39]. Only 2541 participants were included in the analysis (2 participants were excluded due to missing data). Included studies: (1) were completed in the last 10 years (2012–2021), (2) used the CHRT-SR as a measure to assess suicidality of patients at baseline, prior to treatment, and (3) captured overdose events via the adverse event form, death form, and/or hospital utilization form. Trial characteristics are described in Table 1. While each of these eight multisite trials secured approval from their respective Institutional Review Board, the current study only used de-identified data and therefore was exempt from ethical review.

### 2.2. Independent Variable: Suicidality

The independent variable analyzed as the predictor was the baseline suicidality score as measured by the 12-item CHRT-SR [24] that evaluates suicide propensity, ideation, and intent. Items assess signs and symptoms, including characteristics such as pessimism, lack of social support, helplessness, and despair; the last three items assess active suicidal ideation and behavior. Responses are measured by a 5-point Likert scale ranging from strongly disagree (1) to strongly agree (5), with total scores ranging from 12 to 60. The CHRT-SR was also used to create a binary indicator of suicidality if a participant responded “Yes” to any of the three following items indicative of suicidal thoughts: “I have been having thoughts of killing myself”, “I have thoughts about how I might kill myself”, or “I have a plan to kill myself”. The Cronbach’s alpha for CHRT-SR was acceptable for all trials (CTN0037: 0.86; CTN0049: 0.91; CTN0051: 0.87; CTN0053: 0.89; CTN0054: 0.87; CTN0064: 0.90; CTN0067: 0.86; CTN0068: 0.89).

### 2.3. Dependent Variable and Adjudication Process: Overdose Events

An overdose event during the study period, the outcome of interest, was defined as a binary outcome (Present: yes/Absent: no). Responses were determined through a review of the dataset for: (1) MedDRA-Preferred Terms captured in Adverse Event forms, (2) primary cause of death reported on death form, or (3) hospital utilization due to overdose reported on hospitalization forms. Through a rigorous adjudication process, a panel of experts consisting of a subgroup of study co-authors (VEH, RDS, DB, KH, JF, ST) followed explicit steps and key terms recommended by a CTN medical monitor (RK) as they reviewed the recorded adverse events, deaths, and hospitalizations. The key recommended term for adverse event forms was “overdose”, but related terms such as acute amphetamine toxidrome, respiratory depression and drug intoxication were considered where the term overdose was not recorded but suspected. For these overdose suspected cases, the medical monitor reviewed narratives from the study forms for an indication of involved substances which were then discussed by the panel to reach consensus on the adjudication of the outcome. For the 2 trials where both the hospitalization and death forms were used (CTN 0049 [33,41] and CTN 0064 [37,45]), the panel reviewed all causes for hospitalization (primary discharge diagnosis) and the primary cause of death as recorded in the database. Deaths due to overdose were listed as “Drug Use/Overdose” or “Substance Use”. Key terms used to search the hospitalization events included “Overdose”, “Abuse”, “Intoxication”, and “Detox”; however, all primary discharge diagnoses were considered individually by the panel.

### 2.4. Covariates

The panel used a process of consensus to decide on the pertinent covariates to include in the model, and the measures that should be used in each trial to ascertain the variables/covariates of interest. Decisions were made based on the literature and standard practice. Demographic covariates considered across the protocols included age, gender, and race/ethnicity.

Given the correlation between depression and suicidality, the prevalence of baseline depression was also assessed by creating a binary indicator of depression using the instrument that had been part of each trial’s procedures. Depression measures included the 9-item Patient Health Questionnaire (PHQ-9 [26]: CTN0067 [38,46], CTN0068 [39,47]), the 18-item Brief Symptom Inventory (BSI-18 [48]: CTN0049 [33,41], CTN0064 [37,45]), the Addiction Severity Index Lite (ASI Lite [49];CTN0037 [32,40], CTN0051 [34,42]), the Medical and Psychiatric History (CTN0054 [36,44]), and the Hospital Anxiety and Depression Scale (HADS [50]: CTN0053 [35,43]). Because lifetime heroin use [51], recent alcohol and benzodiazepine use [52], and past psychiatric history [53], increase risk of overdose, these factors were also included as covariates in the model. Each of these covariates was assessed by creating binary variables of each distinct instrument or question across the 8 trials. The assessment of lifetime use of heroin included the Addiction Severity Index Lite (ASI Lite [49]: 0037 [32,40], 0049 [33,41], 0051 [34,42], 0064 [37,45], 0067 [38,46]) and the Alcohol and Substance History (0054 [36,44], 0068 [39,47]). One trial, CTN 0054, did not assess lifetime use of heroin. Recent alcohol and benzodiazepine use was determined by creating a binary indicator using each trial’s instrument to assess this variable. The assessment of alcohol and benzodiazepine use was determined by the ASI Lite (0037 [32,40], 0049 [33,41], 0051 [34,42], 0064 [37,45], 0067 [38,46]), and the DSM-5 checklist [54] (CTN0054 [36,44], CTN0053 [35,43], CTN0068 [39,47]). Psychiatric history exclusive of depression was determined as a binary indicator, using each trial’s instrument to assess this variable. The assessment of psychiatric history included the Medical History Form (0051 [34,42], 0054 [36,44], 0067 [38,46], 0068 [39,47]), ASI Lite (CTN 0037 [32,40]), Additional Psychiatric Diagnosis Form (CTN0064 [37,45]), and the Mini International Neuropsychiatric Interview, version 6.0 (MINI 6.0 [55]: 0053 [35,43]). For participants in CTN0049, psychiatric diagnosis was considered via two instruments. First, the team reviewed the Initial Hospital Admission form, and included patients as having a history of psychiatric diagnosis if the primary diagnosis during admission and/or any comorbid diagnoses included terms or conditions such as “suicidal ideation”, “psychosis”, “schizophrenia”, “bipolar disorder”, “PTSD”, “hallucinations”, “mood disorder” and “altered mental state”. Second, if CTN0049 participants reported that they saw a professional for the primary purpose of getting help for psychological or emotional issues in the past 6 months (Service Utilization Detail Form [33,41]), they were also included as having a history of a mental health diagnosis.

Because these trials were diverse in the study treatments, settings, targeted substance use disorders and specific populations, we included trial as a covariate to account for this variability. Treatment arm (experimental or control) was also included to account for the difference in treatments within trials.

### 2.5. Analytic Plan

Descriptive statistics, including mean and standard deviation for continuous variables and frequencies and proportions for categorical variables, were calculated across trials and among participants with and without suicidality and with and without overdose events. A multivariate logistic regression, using a generalized estimating equation, analyzed continuous CHRT-SR score at baseline as a predictor of binary overdose event (present/not present), while controlling for covariates. Adjusted odds ratios and 95% confidence intervals were calculated. For all analyses, two-tailed *p*-values less than 0.05 were considered statistically significant. All analyses were performed using SAS version 9.4 [56].

## 3. Results

A total of 2541 participants were included in this analysis. The majority of participants were male (67.4%) and the mean age was 39.4 years (SD 11.4); 38.3% were Black, 41.3% White, and 14.4% Hispanic. Recent use of alcohol and benzodiazepines was reported by 60.0% and 15.8%, respectively, and 39.0% reported lifetime use of heroin. With regard to co-occurring mental health conditions, 51.6% scored in the depressed range at baseline, and 50.2% indicated that they had at least one preexisting psychiatric diagnosis. The mean baseline CHRT-SR score was 23.9 (SD 8.5; min 11, max 59). A total of 122 (4.8%) either agreed or strongly agreed with the last three items in the CHRT- SR scale and were categorized as suicidal at baseline. Among those who were suicidal, there was a higher proportion of Black/African American (45.1%), followed by White (38.5%) and Hispanic (13.1%) populations. Seventy-five participants (3.0%) had at least one overdose event during their study participation. Of these 49.3% were white, 26.7% were Black/African American and 20.0% were Hispanic. Of those participants who reported suicidal thoughts and intent, only 6 (4.9%) had an overdose event.

**Figure 1:**
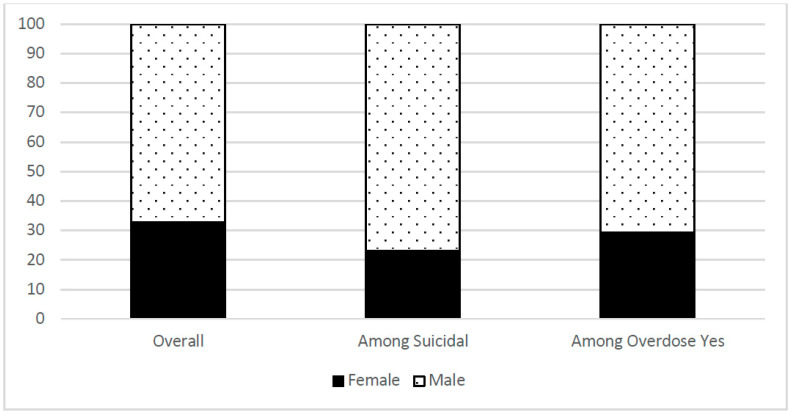
Proportion of Female/Male overall, among those who are suicidal, and among those with an overdose event.

**Figure 2:**
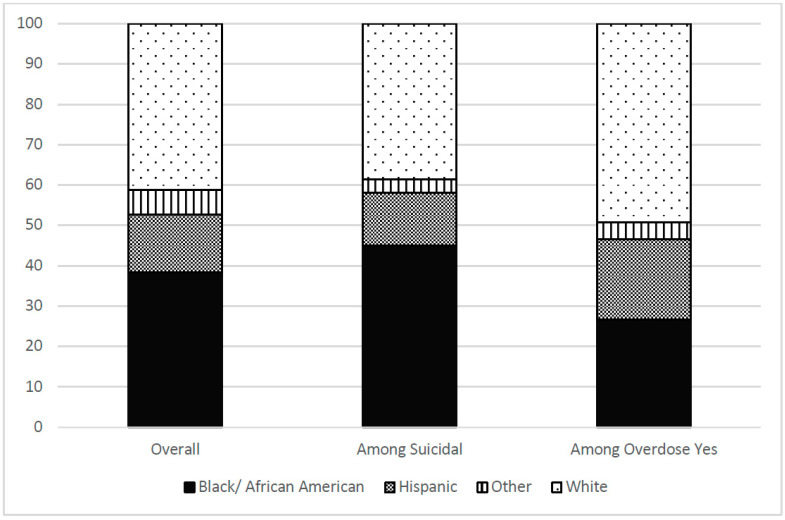
Proportion of Race/Ethnicity overall, among those who are suicidal, and among those with an overdose event.

Demographic characteristics of the participants can be found in Table 2, and proportions of gender and of race/ethnicity overall, among those who were suicidal, and among those with an overdose event can be found in Figure 1 and Figure 2. Demographic information by study can be found in the primary outcomes’ publications [32,33,34,35,36,37,38,39]. Total participants in analyses varied slightly due to occasional missing data. 

Preliminary results of model fit revealed that age, gender, and race/ethnicity were not significant in the model, and therefore dropped from the final model. Results of logistic regression show that the continuous CHRT-SR score was associated (*p* = 0.03) with overdose events and the likelihood of overdose increased as the continuous CHRT-SR score increased (OR 1.02; 95% CI = 1.00–1.04). Depression, recent use of alcohol or benzodiazepines, history of psychiatric disorders, and treatment arm were not associated with higher odds of overdose, but lifetime use of heroin was associated (*p* < 0.01) with increased odds of overdose (OR 3.08; 95% CI = 1.93–4.92). Model results can be found in Table 3.

## 4. Discussion

Results of this study demonstrate that suicide propensity, ideation and intent, as assessed by the continuous CHRT-SR score, were associated with overdose events amongst patients seeking treatment in eight clinical trials for SUD. Increases in the total score of CHRT-SR were associated with a higher likelihood of experiencing an overdose event. To our knowledge this is the first study exploring the relationship of suicidality and overdose events in a substance-using, treatment-seeking population.

Surprisingly, only 4.8% of the sample endorsed suicidal ideation or intent at baseline. This is possibly explained by the fact that most studies excluded actively suicidal patients at enrollment. Suicidal ideation and intent have not been previously documented systematically during treatment in patients with SUD or dual disorders. Rather, assessments have been based on suicide mortality in these populations. Esang and Ahmed [57] reported an increased risk of suicide in patients with substance use and psychiatric conditions. Increased opioid overdose risk has been described in patients with OUD [58]. Studies assessing suicidal intent after an overdose report highly variable prevalence of suicidal ideation. For example, while one study documented that 58.0% of patients with OUD who overdosed indicated a desire to die [18], another reported only 6.6% of opioid overdose survivors firmly expressed their intent to die [16]. In addition, these retrospective studies relied on patients’ self-reports, where reconstruction of the feelings, thoughts, and desire prior to the overdose event might be misconstrued or be prone to recall bias. Suicidal ideation and intent have been documented in prior studies of patients with acute major depression with non-psychotic features. Based on the type of assessment used, the prevalence of suicidal thoughts and intent ranged from 10.7% to 19.0% at baseline [59]. The prevalence of suicidal ideation or intent documented in our study is therefore lower than that observed in patients in treatment for major depression. Of note, the prevalence of depression in our sample was 51.6%, and 50.2% of the participants reported a previous psychiatric history. While the association between depression and SUD in our sample was high, other studies have demonstrated even higher levels of depression in patients with SUD [60].

Results from this study confirm that lifetime heroin use is associated with an increased likelihood of an overdose event. Brandt and colleagues [58] and Stover and colleagues [19] have demonstrated increased risk of opioid overdose events in patients with a history of heroin use. Surprisingly, and in contrast with the literature, neither depression nor recent alcohol or benzodiazepine use were associated with overdose events in our study. This might be explained by the variability in the approach to the assessment of depression across trials, or that participants were only experiencing mild depressive disorders, and therefore, the association with increased overdose was not observed as expected.

Consistent with the literature, overdose events were more frequent in the White population (49.3%), followed by Black/African American (26.7%) and Hispanic (20.0%) populations, whereas suicidality was more frequent in Black/African American (45.1%), followed by White (38.5%) and Hispanic (13.1%) populations. Gicquelais and colleagues [16] found that active or passive suicidal intent was more prevalent in Whites (86.3%) followed by multiracial individuals (9.3%), whereas only 4.3% of the Black/African American sample intended to die. In our study, the choice of substance used may have accounted for the racial/ethnic differences we found. However, this is speculative, given that most of the information on the overdose was self-reported at the time of documentation and was inconsistently documented in the narrative of overdose events, preventing identification of specific substances involved.

The present study has several strengths. First, it provides an examination across multiple sites and multiple studies expanding on prior single site examinations of suicidality and overdose. Second, it expands on the use of large datasets to rapidly answer practical clinical questions and furthers the understanding of mental health conditions and their association with adverse substance use outcomes.

This study also presents several limitations. First, the design of this study only allowed for examination of associations and not causation. Second, the population enrolled across these eight clinical trials is representative of individuals seeking treatment for SUD who agreed to participate in a research study. Therefore, our results might not be generalizable to the entire population of persons with SUD and other co-occurring mental health conditions. It is noteworthy that while the panel of experts ascertained outcomes using a binary yes/no approach for each of the covariates, the measures used to evaluate these variables differed across trials and may have had slightly different meanings. However, the tradeoff of this heterogeneity might be mitigated by the large sample size accrued across multiple sites and conducted over 10 years. Finally, heroin use was not assessed in one of the trials and is reported as missing data in our results.

This study highlights the relevance of assessing suicidality at baseline for patients entering treatment. As drug overdose deaths continue to rise in the U.S. and worldwide and suicide remains a leading cause of death, particularly in patients with comorbid substance abuse and mental health disorders, multi-pronged approaches covering prevention, early detection, and intervention are needed. Integration of suicide risk reduction should be included as part of an overall prevention strategy in response to the steep climb in overdose fatalities. Entry into treatment for SUD and other co-occurring mental health conditions presents an invaluable opportunity to re-set, evaluate suicide risk, and employ early prevention plans.

## Figures and Tables

**Table 1:** Selected CTN Trial Characteristics.

Trial	Study Title	Study Type	Sample Size	Main Target Substance	Recruitment Setting	Intervention Period/Follow Up Period
CTN 0037 [32,40]	Stimulant Reduction Intervention Using Dosed Exercise (STRIDE)	2-arm RCT	302	Stimulants (Cocaine and Methamphetamine)	Residential substance use treatment programs	12 weeks/ 36 weeks
CTN 0049 [33,41]	Project HOPE: Hospital Visit as Opportunity for Prevention and Engagement for HIV-Infected Drug Users	3-arm RCT	801 *	Any substance	Inpatient, Hospitalized, enrolled at bedside	26 weeks/ 52 weeks
CTN 0051 [34,42]	Extended-Release Naltrexone vs. Buprenorphine for Opioid Treatment (X:BOT)	2-arm comparative effectiveness RCT	570	Opioids	Community based treatment programs	24 weeks/ 36 weeks
CTN 0053 [35,43]	Achieving Cannabis Cessation: Evaluating N-Acetylcysteine Treatment (ACCENT)	Double-blind, placebo controlled 2-arm RCT	302	Cannabis	Multicenter, “treatment-seeking cannabis-dependent adults who submit positive urine cannabinoid testing during screening”	12 weeks/ 16 weeks
CTN 0054 [36,44]	Accelerated Development of Additive Pharmacotherapy Treatment (ADAPT)	2-stage pilot study	49	Methamphetamine	Outpatient, community treatment programs	8 weeks/ 9 weeks
CTN 0064 [37,45]	Linkage to Hepatitis C Virus (HCV) Care among HIV/HCV Co-infected Substance Users	2-arm RCT	113	Any substance	Follow up population of CTN 0049	26 weeks/ 52 weeks
CTN 0067 [38,46]	Comparing Treatments for HIV-Infected Opioid Users in an Integrated Care Effectiveness Study (CHOICES) Scale-Up	2-arm RCT	116	Opioids	Primary Care	24 weeks/ 24 weeks
CTN 0068 [39,47]	Accelerated Development of Additive Pharmacotherapy Treatment (ADAPT-2) for Methamphetamine Use Disorder	Double-blind, placebo controlled 2-arm RCT with adaptive design	403	Methamphetamine	Adults 18–65 were recruited from communities near the trial sites with the use of ads and direct referrals	12 weeks/ 16 weeks

RCT: Randomized Controlled Trial. * The CTN 0049 population assessed in this analysis includes only those unduplicated patients (*N* = 688 of 801 total) who were not re-randomized to CTN 0064 (*N* = 113).

**Table 2:** Participant characteristics overall, by suicidal yes/no, by overdose yes/no.

		Overall (*N* = 2541)	Suicidal * (*N* = 122)	Non-Suicidal (*N* = 2418)	Mean CHRT-SR Score	Yes Overdose (*N* = 75)	No Overdose (*N* = 2466)
		Mean (Standard Deviation) or *N* (%)	M (SD) or *N* (%)	M (SD) or *N* (%)	M (SD)	M (SD) or *N* (%)	M (SD) or *N* (%)
Age		39.4 (11.4)	42.4 (10.5)	39.2 (11.5)	-	39.1 (11.8)	39.4 (11.4)
Sex	Female	829 (32.6%)	28 (23.0%)	800 (33.1%)	23.8 (8.3)	22 (29.3%)	806 (32.7%)
	Male	1712 (67.4%)	94 (77.0%)	1618 (66.9%)	23.9 (8.6)	53 (70.7%)	1659 (67.3%)
Race/Ethnicity	Black/Af Am	972 (38.3%)	55 (45.1%)	916 (37.9%)	23.5 (8.6)	20 (26.7%)	951 (38.6%)
	Hispanic	366 (14.4%)	16 (13.1%)	350 (14.5%)	24.0 (8.4)	15 (20.0%)	351 (14.2%)
	Other	153 (6.0%)	4 (3.3%)	149 (6.2%)	25.0 (8.0)	3 (4.0%)	150 (6.1%)
	White	1050 (41.3%)	47 (38.5%)	1003 (41.5%)	23.9 (8.6)	37 (49.3%)	1013 (41.1%)
Treatment Arm Assignment	Experimental	1310 (51.6%)	64 (52.5%)	1245 (51.5%)	24.0 (8.5)	45 (60.0%)	1265 (51.3%)
	Control	1231 (48.5%)	58 (47.5%)	1173 (48.5%)	23.7 (8.6)	30 (40.0%)	1200 (48.7%)
Depressed	Yes	1310 (51.6%)	112 (91.8%)	1197 (49.5%)	27.0 (8.7)	37 (49.3%)	1273 (51.7%)
	No	1230 (48.4%)	10 (8.2%)	1220 (50.5%)	20.5 (6.9)	38 (50.7%)	1191 (48.3%)
History of Psychiatric Diagnosis	Yes	1276 (50.2%)	75 (61.5%)	1200 (49.6%)	25.2 (8.7)	42 (56.0%)	1233 (50.0%)
	No	1265 (49.8%)	47 (38.5%)	1218 (50.4%)	22.5 (8.1)	33 (44.0%)	1232 (50.0%)
Recent Alcohol Use	Yes	1523 (60.0%)	72 (60.0%)	1450 (60.0%)	23.5 (8.6)	37 (49.3%)	1485 (60.3%)
	No	1016 (40.0%)	48 (40.0%)	968 (40.0%)	24.4 (8.4)	38 (50.7%)	978 (39.7%)
Recent Benzo Use	Yes	400 (15.8%)	19 (15.8%)	381 (15.8%)	25.2 (8.6)	18 (24.0%)	382 (15.5%)
	No	2139 (84.2%)	101 (84.2%)	2037 (84.2%)	23.6 (8.5)	57 (76.0%)	2081 (84.5%)
Lifetime Heroin Use	Yes	992 (39.0%)	41 (33.6%)	951 (39.3%)	25.4 (8.0)	53 (70.7%)	939 (38.1%)
	No	1245 (49.0%)	74 (60.7%)	1170 (48.4%)	23.8 (8.9)	21 (28.0%)	1223 (49.6%)
	Missing	304 (12.0%)	7 (5.7%)	297 (12.3%)	19.1 (7.0)	1 (1.3%)	303 (12.3%)
Suicidal	Yes	122 (4.8%)	122 (4.8%)	-	39.8 (7.9)	6 (8.0%)	116 (4.7%)
	No	2418 (95.2%)	-	2418 (95.2%)	23.1 (7.8)	69 (92.0%)	2348 (95.2%)
Overdose	Yes	75 (3.0%)	6 (4.9%)	69 (2.9%)	25.8 (8.8)	75 (3.0%)	-
	No	2465 (97.0%)	116 (95.1%)	2348 (97.1%)	23.8 (8.5)	-	2465 (97.1%)
CHRT-SR Score		23.9 (8.5)	39.8 (7.8)	23.1 (7.8)	-	25.8 (8.8)	23.8 (8.5)

* One participant is missing from the suicidal total due to missing responses to the last 3 items on CHRT.

**Table 3:** Results of logistic regression/generalized estimating equation assessing CHRT-SR as a continuous predictor of overdose.

		Odds Ratio	95% Confidence Limits		*p*-Value
CHRT-SR Score		1.02	1.00	1.04	0.03
Depressed	Yes	0.76	0.32	1.83	0.54
	No	0	0	0	
Recent Alcohol Use	Yes	0.81	0.63	1.05	0.11
	No	0	0	0	
Recent Benzo Use	Yes	1.40	0.77	2.54	0.27
	No	0	0	0	
Lifetime Heroin Use	Missing	0.19	0.11	0.32	<0.01
	Yes	3.08	1.93	4.92	<0.01
	No	0	0	0	
History of Psychiatric Diagnosis	Yes	0.85	0.65	1.11	0.23
	No	0	0	0	
Treatment Arm	Experimental	1.48	0.81	2.70	0.20
	Control	0	0	0	.

## Data Availability

CTN trial data is publicly available on NIDA Data Share: https://datashare.nida.nih.gov/ (accessed on 15 February 2022). CTN 0064, CTN 0067 and 0068 data has been approved for upload onto NIDA Data Share and will be publicly available soon.
